# The Adenylyl Cyclase Inhibitor MDL-12,330A Potentiates Insulin Secretion via Blockade of Voltage-Dependent K^+^ Channels in Pancreatic Beta Cells

**DOI:** 10.1371/journal.pone.0077934

**Published:** 2013-10-29

**Authors:** Xiaodong Li, Qing Guo, Jingying Gao, Jing Yang, Wan Zhang, Yueqin Liang, Dongmei Wu, Yunfeng Liu, Jianping Weng, Qingshan Li, Yi Zhang

**Affiliations:** 1 Department of Pharmacology, Shanxi Medical University, Taiyuan, China; 2 Department of Endocrinology, the First Hospital of Shanxi Medical University, Shanxi Medical University, Taiyuan, China; 3 Department of Endocrinology, the Third Affiliated Hospital of Sun Yat-Sen University, Guangzhou, China; 4 School of Pharmaceutical Sciences, Shanxi Medical University, Taiyuan, China; Vanderbilt University Medical Center, United States of America

## Abstract

**Objective:**

Adenylyl cyclases (ACs) play important role in regulating pancreatic beta cell growth, survival and secretion through the synthesis of cyclic AMP (cAMP). MDL-12,330A and SQ 22536 are two AC inhibitors used widely to establish the role of ACs. The goal of this study was to examine the effects of MDL-12,330A and SQ 22536 on insulin secretion and underlying mechanisms.

**Methods:**

Patch-clamp recording, Ca^2+^ fluorescence imaging and radioimmunoassay were used to measure outward K^+^ currents, action potentials (APs), intracellular Ca^2+^ ([Ca^2+^]_i_) and insulin secretion from rat pancreatic beta cells.

**Results:**

MDL-12,330A (10 µmol/l) potentiated insulin secretion to 1.7 times of control in the presence of 8.3 mmol/l glucose, while SQ 22536 did not show significant effect on insulin secretion. MDL-12,330A prolonged AP durations (APDs) by inhibiting voltage-dependent K^+^ (K_V_) channels, leading to an increase in [Ca^2+^]_i_ levels. It appeared that these effects induced by MDL-12,330A did not result from AC inhibition, since SQ 22536 did not show such effects. Furthermore, inhibition of the downstream effectors of AC/cAMP signaling by PKA inhibitor H89 and Epac inhibitor ESI-09, did not affect K_V_ channels and insulin secretion.

**Conclusion:**

The putative AC inhibitor MDL-12,330A enhances [Ca^2+^]_i_ and insulin secretion via inhibition of K_V_ channels rather than AC antagonism in beta cells, suggesting that the non-specific effects is needed to be considered for the right interpretation of the experimental results using this agent in the analyses of the role of AC in cell function.

## Introduction

Adenylyl cyclase (AC) is a crucial enzyme that catalyses the synthesis of cyclic AMP (cAMP) from ATP. As an ubiquitous second messenger, cAMP plays key roles in a variety of fundamental cell functions ranging from cell growth and differentiation, to transcriptional regulation and apoptosis [1]–[3]. The effects of cAMP are mediated by two downstream effectors, protein kinase A (PKA) and exchange protein directly activated by cAMP (Epac) [4]. In pancreatic beta cells, AC/cAMP signaling pathway is known important in regulating beta cell growth, survival and glucose-induced insulin secretion [5], [6]. cAMP is also a pivotal component that mediates the functions of some insulinotropic hormones, such as glucagon-like peptide-1 (GLP-1) and glucose-dependent insulinotropic polypeptide (GIP) [7], [8].

For investigating the role of AC/cAMP signaling pathway, pharmacological tools have been chosen to modulate AC activities in many studies. Among which, MDL-12,330A is one of the most widely used agents as a specific AC inhibitor [9]. However, in the present study, the non-specific effect of MDL-12,330A on K_V_ channels has been observed in pancreatic beta cells.

Pancreatic beta cells are electrically excitable cells that secrete insulin to maintain blood glucose homeostasis. A number of ion channels contribute to this function. Among these channels, the closure of ATP-sensitive K^+^ channels (K_ATP_ channels) initiates membrane depolarization at high glucose and the voltage dependent Ca^2+^ channels play a key role for action potential firing and insulin secretion [10]. Voltage-dependent K^+^ channels (K_V_) are involved in the repolarization phase of the action potential. It has been shown that blockade of the K_V_ channel prolongs action potential duration (APD) and enhances insulin secretion from beta cells [11], [12]. Here we report that in pancreatic beta cells, MDL-12,330A potently blocks K_V_ channels, extends APD, and enhances insulin secretion. In contrast, similar effects were not observed using another widely used AC inhibitor SQ 22536, or PKA inhibitor H89, or Epac inhibitor ESI-09, implying that the non-specific effects is needed to be considered for the right interpretation of the experimental results using MDL-12,330A, in the study of AC function.

## Materials and Methods

### Animals

Adult male Sprague–Dawley (SD) rats, weighing 250–300 g, were purchased from the Animal Facility Center of Shanxi Medical University. Rats were housed with food and water available ad libitum. under conditions of 23±3°C with a 12 h-light/dark cycle. All protocols and procedures of our experiments described below were approved by the Animal Care and Use Committee of the Shanxi Medical University (Taiyuan, PR China), and all efforts were made to minimize the number of animals used and their suffering, in accordance with the ethical guidelines for animal research in Shanxi Medical University.

### Islet Isolation and Cell Culture

Pancreatic islets were isolated from male SD rats by collagenase p (Roche, Indianapolis, IN, USA) digestion and separated by density gradient centrifugation using histopaque as described previously [13]. Single islet cells were dispersed from rat islets by Dispase II digestion for 6 min. Intact islets or dispersed islet cells were maintained in Hyclone RPMI 1640 (Hyclone Beijing, China) medium containing 11.1 mmol/l glucose supplemented with 10% fetal bovine serum, 0.004% β-mercaptoethanol, 100 U/ml penicillin and 100 µg/ml streptomycin, at 37°C in an atmosphere of humidified air (95%) and CO_2_ (5%) [14]. Animal procedures were performed in accordance with the Shanxi Medical University’s Animal Care Committee’s ethical guidelines.

### Electrophysiology

Islet cells were cultured on glass coverslips for 24 h in medium containing 11.1 mM glucose prior to experiments and were patch-clamped at room temperature. Voltage-clamp and current-clamp experiments were carried out using the Giga-seal patch-clamp method. The beta cells were identified by cell size (>4 pF) [15]. The measurements were performed using an EPC-10 amplifier for the recording of K_V_ currents or Ca^2+^ currents and digitized with PULSE software from HEKA Electronik (Lambrecht, Germany). Patch pipettes were pulled from 1.5 mm outside diameter and 0.84 mm inside diameter borosilicate glass tubes using a two-stage Narishige MODEL PP-830 micropipette puller (Narishige Co., Tokyo, Japan) and fire polished by MIRO FORGE MF-830 to resistances of 4–6 MΩ. Then filled with intracellular solution for K_V_ current recordings containing (mmol/l): 140 KCl; 10 NaCl; 1 MgCl_2_; 0.05 EGTA; 10 HEPES; 0.3 MgATP; pH 7.3 adjusted with KOH. K_V_ extracellular solution contained (mmol/l): 141.9 NaCl; 1.2 MgCl_2_; 5 HEPES; 5.6 KCl; 11.1 glucose; pH 7.4 adjusted with NaOH. In the recordings of depolarization-evoked Ba^2+^ currents, the following intracellular solution was used (mmol/l): 120 CsCl; 20 Tetraethylammonium chloride (TEA); 1 MgCl_2_; 0.05 EGTA; 10 HEPES; and 5 MgATP; pH 7.3, with CsOH. Extracellular solution containing (mmol/l): 100 NaCl; 20 TEA; 20 BaCl_2_; 5 HEPES; 4 CsCl; 1 MgCl_2_; and 11.1 glucose; pH 7.4 adjusted with NaOH. Here, we employed Ba^2+^ to replace Ca^2+^ as the charge carrier to amplify current responses through Ca^2+^ channels during the stimulus of depolarization. In current-clamp mode, action potentials were elicited by applying 4 ms, 150 pA current pulse injections. APD was calculated as the difference in the time from initiation of the action potential until the time that the membrane potential returned to within 10 mV of the resting membrane potential [16]. Data were acquired with Pulsefit v8.78 software (HEKA Electronik). Inhibition of K_V_ current by pharmacological agents was calculated using the Sigmaplot version 12.0 using the four-parameter logistic equation of the following form: Fraction blocked = min+(max−min)/(1+(x/IC_50_)^−Hillslope^), where IC_50_ is the concentration that produces 50% of the blockage.

### Ca^2+^ Fluorescence Imaging

Primary pancreatic islet cells were grown in 35 mm dishes and allowed to attach to the dishes’ surface for 24 h in RPMI 1640 medium with 10% FBS. The cells were then loaded with 4 µM Fluo 4-AM (Dojindo Laboratories, Japan) diluted in low-glucose (2.8 mmol glucose) KRB buffer solution containing the following ingredients (mmol/l): 128.8 NaCl; 4.8 KCl; 1.2 KH_2_PO_4_; 1.2 MgSO4; 2.5 CaCl_2_; 5 NaHCO_3_; 10 HEPES; 2% BSA at pH 7.4 and incubated for 30 min at 37°C with 5% CO_2_. After this, cells were washed twice using low-glucose KRB solution without Fluo 4-AM. [Ca^2+^]_i_ measurements were carried out using OLYMPUS FV1000 (Inverted microscope IX81) confocal microscope at room temperature. Fluo 4-AM was excited at 494 nm and emission was collected at 516 nm. The ratio of fluorescence change F/F_0_ (Cell fluorescence was subtracted from background and autofluorescence) was plotted to represent the change in intracellular Ca^2+^ levels.

### Measurements of Insulin Secretion in Rat Pancreatic Islets

Insulin secretion from rat islets was measured using Iodine [^125^I] Insulin Radioimmunoassay Kit (North biological technology research institute of Beijing). Rat islets were cultured for 24 h at 37°C and 5% CO_2_, then 5 islets per well were pre-incubated in Krebs-Ringer bicarbonate-HEPES(KRBH) buffer containing (mmol/l): 128.8 NaCl; 4.8 KCl; 1.2 KH_2_PO_4_; 1.2 MgSO_4_; 2.5 CaCl_2_; 5 NaHCO_3_; 10 HEPES; and 2% BSA at pH 7.4 containing 2.8 mmol glucose for 30 min. After pre-incubation, islets were then incubated for 30 min in KRB solution containing 8.3 mM glucose in the presence or absence of MDL-12,330A (Sigma-Aldrich Co. USA), SQ 22536 (Cayman Chemical, Ann Arbor, Mich., USA), H89 (Cayman Chemical, Ann Arbor, Mich., USA) and ESI-09 (Cayman Chemical, Ann Arbor, Mich., USA). Then, the incubated supernatant was collected for insulin concentration measurement. The total insulin contents per well of these islets lysed with 70% acid-ethanol solution (Ethanol/water/HCl (vol./vol.) = 150∶47:3) were measured. All values were normalized to total insulin content. The insulin secretion studies were carried out at 37°C.

### Measurements of cAMP Production in Rat Pancreatic Islets

In cAMP measurements, each tube containing 10 islets was incubated for 1 h in KRBH with 2.8 or 8.3 mmol/l glucose containing 500 µmol/l isobutyl-1-methylxanthine (IBMX) and 100 µmol/l 4-(3-butoxy-4-methoxy-benzyl) imidazolidone (Ro 20–1724). IBMX (Cayman Chemical, Ann Arbor, Mich., USA) and Ro 20–1724 (Sigma-Aldrich) are broad-range phosphodiesterase (PDE) inhibitors to avoid degradation of cAMP in the samples. MDL-12,330A, SQ 22536 or forskolin (Cayman Chemical, Ann Arbor, Mich., USA) were applied during the experiment as indicated. Total cellular cAMP content in islets were determined by RIA kit (Institue of isotopes Co.Ltd, HU).

### Statistical Analysis

All data are presented as mean ± SEM. Statistical analysis was done by Student’s t-test, and significant difference was assumed at p<0.05 using the Sigmaplot version 12.0. Patch clamp data were analyzed by the softwares of IGOR Pro 6.1 (Wavemetrics, Lake Oswego, OR) and PULSEFIT (HEKA Electronik). Laser confocal data were analyzed with FV10-ASW 3.0 software (Olympus Corporation of Japan).

## Results

### MDL-12,330A and SQ 22536 have Similar Effects on cAMP, but Differ in Insulin Secretion

**Figure 1:**
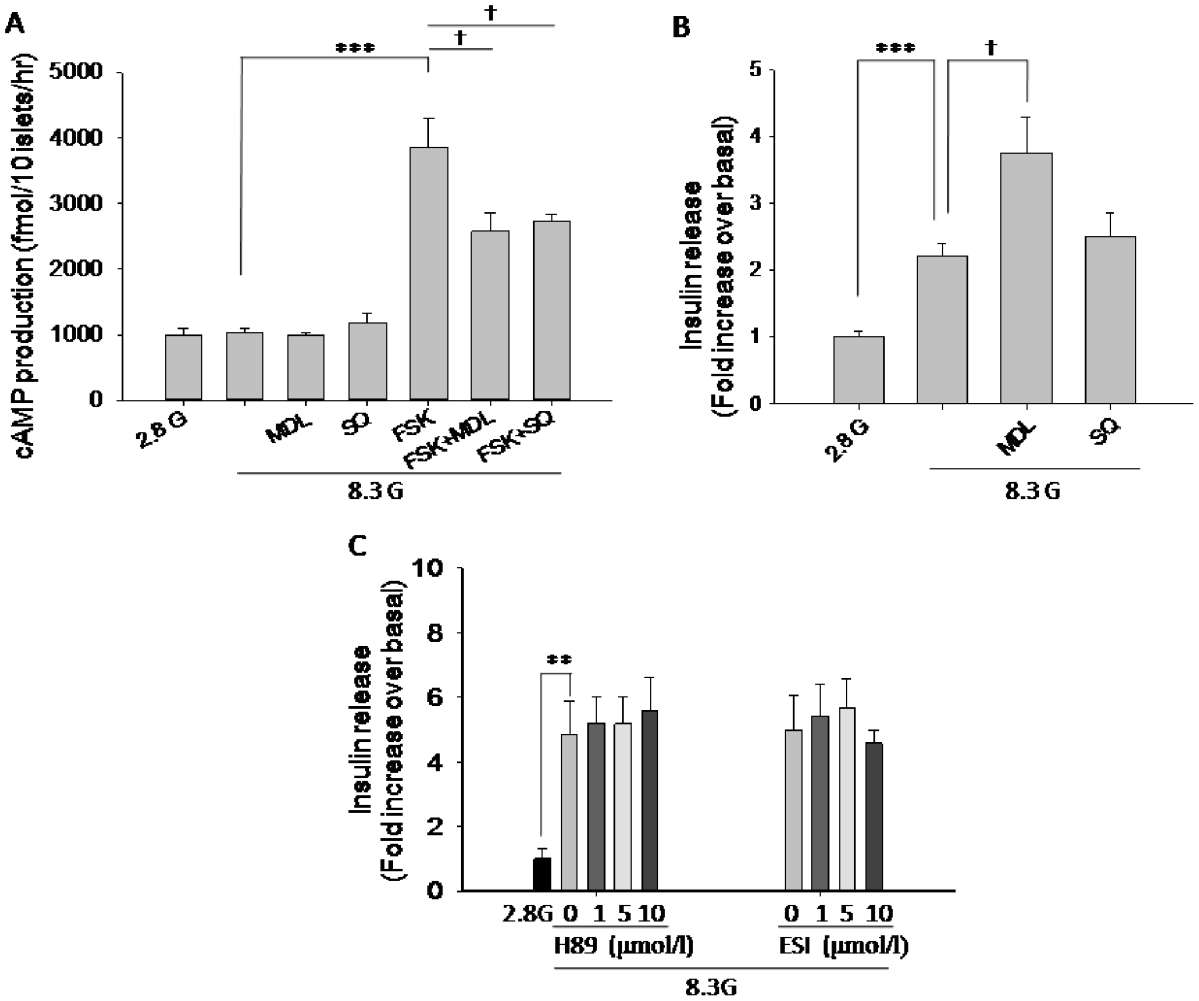
MDL-12,330A and SQ 22536 have similar effects on cAMP, but differ in insulin secretion. A. effects of MDL-12,330A (MDL, 10 µM, n = 6) and SQ 22536 (SQ, 10 µM, n = 6) on intracellular cAMP in the absence or presence of forskolin (FSK, 10 µM ) at 2.8 mM glucose (2.8 G) or 8.3 mM glucose (8.3 G) conditions. B. effects of MDL-12,330A (10 µM, n = 6) and SQ 22536 (10 µM, n = 8) on insulin secretion at 8.3 mM glucose condition. C. effects of H89 (1 µM, n = 8; 5 µM, n = 7; 10 µM, n = 8) and ESI-09 (ESI, 1 µM, n = 6; 5 µM, n = 6; 10 µM, n = 5) on insulin secretion at 8.3 mM glucose condition. **P<0.01, ***P<0.001, ^†^P<0.05.

We first examined the effects of MDL-12,330A and SQ 22536 on cellular cAMP content in rat pancreatic islets. Consistent with the report by Nakazaki et al [17], our data showed that at 2.8 and 8.3 mmol/l glucose conditions, cAMP were at the same level, and application of MDL-12,330A or SQ 22536 did not change cAMP level as well under these conditions. However, MDL-12,330A and SQ 22536 both markedly inhibited forskolin-induced elevation of cAMP (Fig. 1A). To assess the functional influence of MDL-12,330A on pancreatic beta cells, we then tested insulin secretion using isolated rat islets. As shown in Fig. 1B, 8.3 mmol/l glucose stimulated insulin secretion significantly compared to 2.8 mmol/l glucose, and MDL-12,330A (10 µmol/l) further potentiated insulin secretion (n = 6, P<0.05 vs 8.3 G) in the presence of 8.3 mmol/l glucose. We then applied another widely used AC inhibitor, SQ 22536, to see if similar effects on insulin secretion occur. However, under the same condition, SQ 22536 did not exhibit significant effect on insulin secretion. We then wondered whether modulation of cAMP downstream effectors influences insulin secretion. We found that PKA inhibitor H89 and Epac inhibitor ESI-09 [18], both did not cause changes of insulin secretion (Fig. 1C). These data indicate that MDL-12,330A may have distinct role to alter beta cell excitability via the mechanism that is independent of AC/cAMP signaling pathway.

### MDL-12,330A, but not SQ 22536, Produces Prolonged APDs

**Figure 2:**
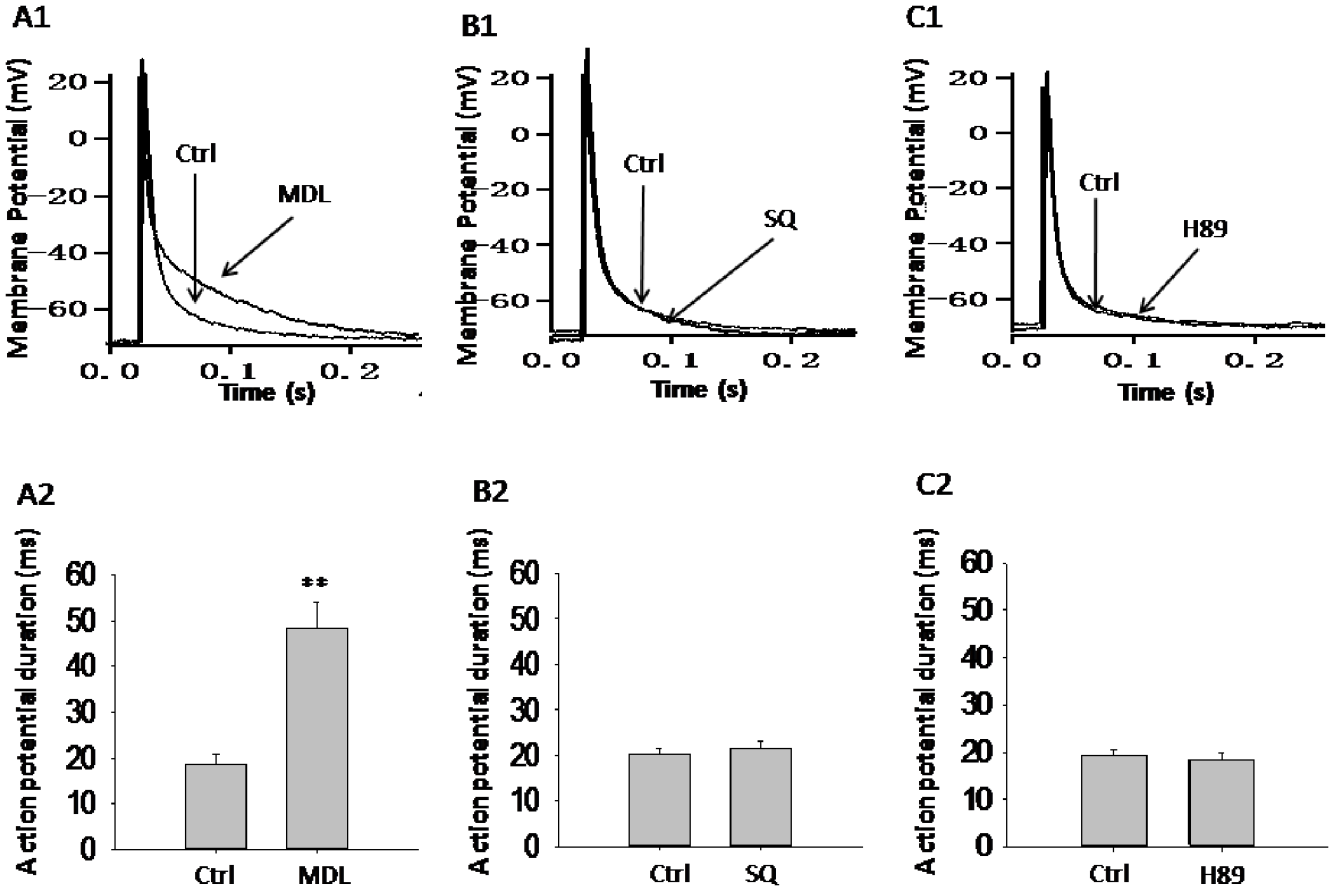
MDL-12,330A, but not SQ 22536, produces prolonged APDs. Pancreatic beta cells were seeded on glass coverslips and cultured in culture dish for 24-clamp experiments. Artificial APs were elicited by applying 4 ms, 150 pA current injections in perforated whole-cell configuration and were recorded before and 2 min after application of reagents in the same cell. Representative action potential waveforms and summary of mean APDs were shown for cells treated with MDL-12,330A (MDL, 10 µM, n = 6), SQ 22536 (SQ, 10 µM, n = 7) or H89 (1 µM n = 7) at 11.1 mM glucose (Ctrl) conditions. **p<0.01.

To examine how MDL-12,330A modulates beta cell excitability, we investigated the effect of MDL-12,330A on electrically induced action potentials. Artificial APs were elicited by applying 4 ms, 150 pA current injections and were recorded before and 2 min after application of reagents in the same cell as shown in Fig. 2. Application of MDL-12,330A remarkably lengthened the APD from 18.5±2.1 ms of control (n = 6) to 48.4±5.7 ms (n = 6, *p*<0.01, Fig. 2A2). However, there were no differences in APDs observed before and after application of SQ 22536 (Fig. 2B2). Moreover, treatment with H89 also did not influence APDs as shown in Fig. 2C2.

### MDL-12,330A Inhibits K_V_ Channels in Pancreatic Beta Cells

**Figure 3:**
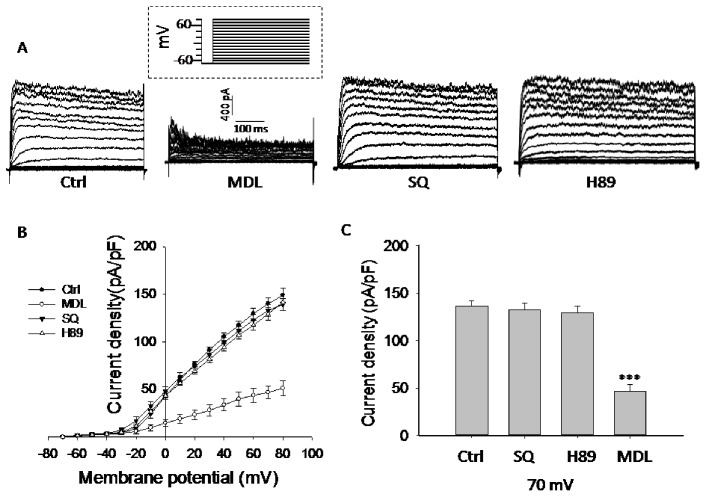
MDL-12,330A inhibits K_V_ channels in pancreatic beta cells. K_V_ currents were recorded in conventional whole-cell configuration from a holding potential of −70 mV to various depolarizing voltages (−70 to 80 mV) as the protocol shown in inset. A. representative current traces recorded under the different treatments as indicated. B. current-voltage relationship curves of K_V_ channels from rat beta cell. C. summary of the mean current density of K_V_ channels recorded at 70 mV depolarization. Control (Ctrl, n = 12); MDL-12,330A (MDL, 10 µM, n = 6); SQ 22536 (SQ, 10 µM, n = 7); H89 (1 µM, n = 7). ***P<0.001.

**Figure 4:**
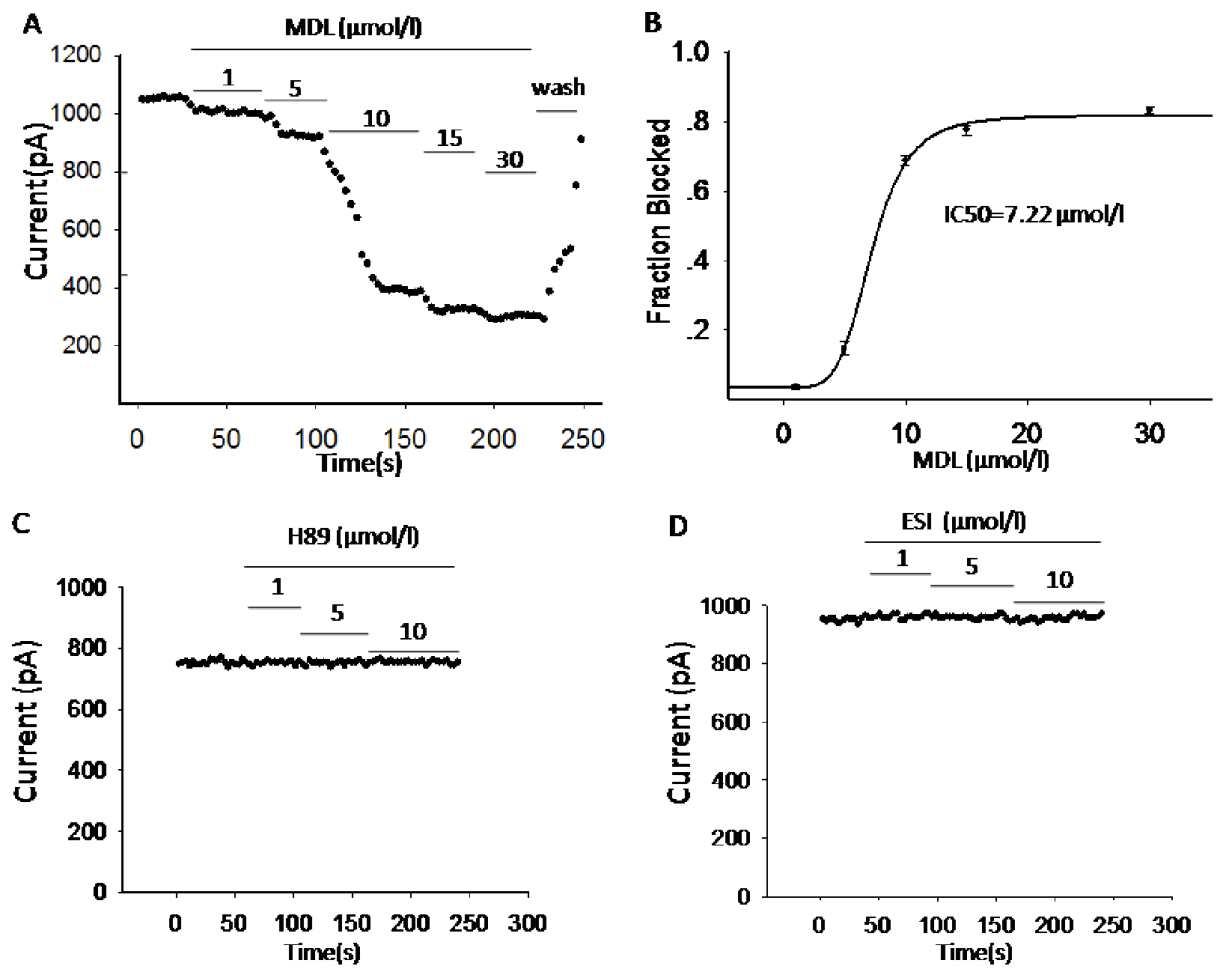
MDL-12,330A inhibits K_V_ currents in a dose-dependent manner in rat beta cells. A. representative time-response curve of K_V_ currents in response to 400 ms voltage steps to +70 mV in the absence and presence of 1, 5, 10, 15 and 30 µM MDL-12,330A (MDL) in the same cell. The holding potential was −70 mV and voltage steps were applied every 3 s in perforated whole-cell configuration. The addition of MDL-12,330A is indicated by the bar with the specific concentrations. B. plot of the fraction of control current blocked (Fraction Blocked) versus MDL-12,330A concentration for all cells tested (n = 5). The continuous line is a fit of the four-parameter logistic equation to the data. Parameters of the fit are: IC_50_ = 7.22 µM. C and D. representative time-response scatterplot of K_V_ currents in the absence and presence of H89 (n = 7) and ESI-09 (ESI, n = 7).

Prolonged APD can result from either decreased repolarizing currents (e.g. voltage-gated K^+^ currents) or increased depolarizing currents (e.g. voltage-dependent Ca^2+^ currents). We therefore first monitored K_V_ currents using whole-cell voltage-clamp technique. The result demonstrated that sustained K_V_ currents were potently inhibited by MDL-12,330A. As shown in Fig. 3B and C, MDL-12,330A (10 µmol/l) only elicited 35% of K_V_ currents compared to control at 70 mV (47.0±6.5 pA/pF for MDL-12,330A, n = 6; 136.5±5.0 pA/pF for Control, n = 12; p<0.001). Consistent with the results from insulin secretion and membrane potential measurements described above, both SQ 22536 (132.5±7.1 pA/pF, n = 7) and H89 (129.5±6.4 pA/pF, n = 7) did not cause changes of K_V_ currents compared to control (Fig. 3B and C). Further study using perforated whole cell technique showed that MDL-12,330A dose-dependently inhibited K_V_ channels; the IC_50_ observed in rat beta cells is 7.22 µmol/l (Fig. 4B). H89 and ESI-09 had no effect on K_V_ currents (Fig. 4C and D). The results imply that the prolonged action potential induced by MDL-12,330A, at least partially, is due to the inhibition of K_V_ channels.

### MDL-12,330A does not Influence Currents through Voltage-dependent Ca^2+^ Channels

**Figure 5:**
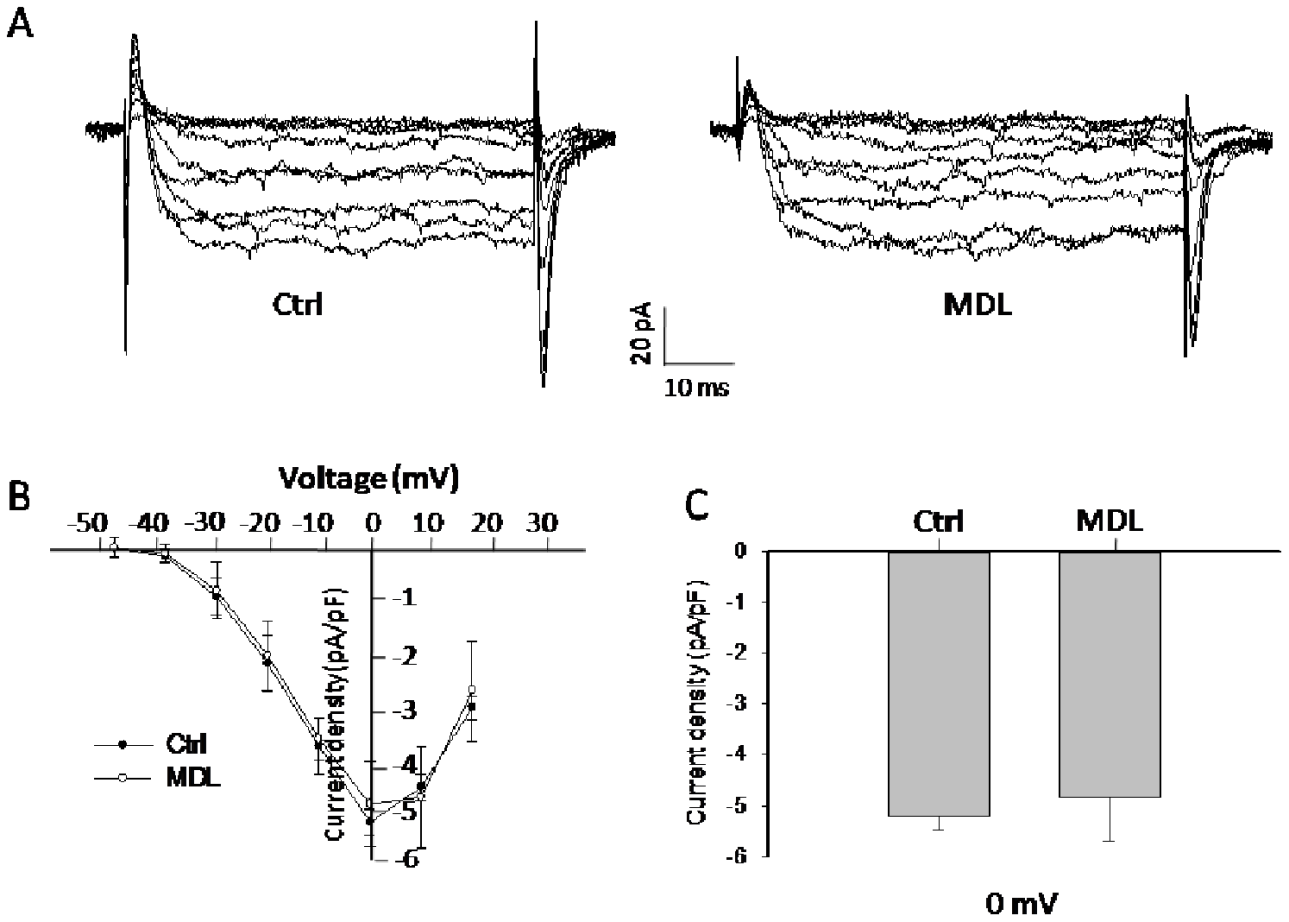
MDL-12,330A does not influence currents through voltage-dependent Ca^2+^ channels. We employed extracellular Ba^2+^ to replace Ca^2+^ as the charge carrier to amplify current responses through Ca^2+^ channels during the stimulus of depolarization. Ba^2+^ currents were recorded in conventional whole-cell configuration from a holding potential of −70 mV to various depolarizing voltages (−50 to 20 mV). A. representative current traces recorded under the treatments as indicated. B. current-voltage relationship curves of Ca^2+^ channels from rat beta cell. C. summary of the mean current density of Ba^2+^ currents recorded at 0 mV depolarization. Control (Ctrl, n = 6); MDL-12,330A (MDL, 10 µM, n = 6).

We next would like to know if voltage-dependent Ca^2+^ channel is also involved in the modulation of APDs induced by MDL-12,330A. Here, we employed extracellular Ba^2+^ to replace Ca^2+^ as the charge carrier to amplify current responses through Ca^2+^ channels during the stimulus of depolarization. As shown (Fig. 5A), depolarizing pulses from potentials of −50 to 20 mV evoked inward Ba^2+^ currents from islet beta cells. The current density was −4.8±0.9 pA/pF (n = 6) for MDL-12,330A at 0 mV, which did not differ from control (−5.2±0.3 pA/pF n = 6, p>0.05, Fig. 5C). The current-voltage relationship also demonstrated that MDL-12,330A did not change the property of Ca^2+^ channels (Fig. 5B). Thus, the prolongation of APD by MDL-12,330A is likely due to the inhibition of K_V_ channel rather than the change of Ca^2+^ channel.

### MDL-12,330A Influences Insulin Secretion via K_V_ Channels Rather than K_ATP_ Channels in Rat Pancreatic Islets

**Figure 6:**
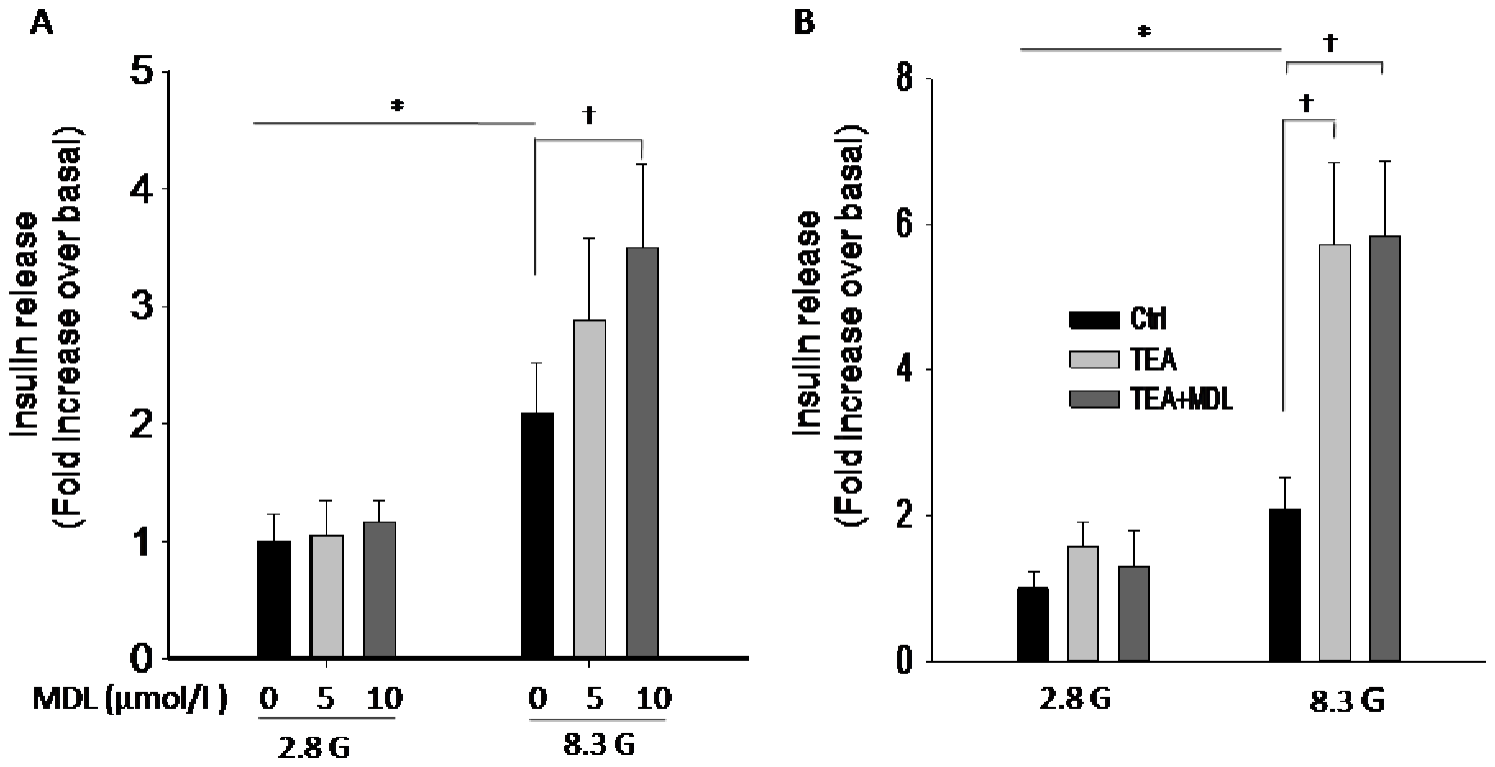
MDL-12,330A influences insulin secretion via K_V_ channels rather than K_ATP_ channels in rat pancreatic islets. All islets were preliminary incubated for 30(2.8 G), then incubated in KRB solution containing 2.8 G or 8.3 G in the presence or absence of the compounds as indicated. Insulin release from these islets was subsequently assayed and the values were normalized to basal secretion at 2.8 G. A. effects of MDL-12,330A on insulin secretion at 2.8 G (MDL 0 µM, n = 6; MDL 5 µM, n = 6; MDL 10 µM, n = 5) and at 8.3 G condition (MDL 0 µM, n = 5; MDL 5 µM, n = 6; MDL 10 µM, n = 5). B. effects of TEA (20 mM) on insulin secretion in the absence or presence of MDL-12,330A (10 µM) at 2.8 G (Ctrl, n = 6; TEA, n = 5; TEA+MDL, n = 5) and at 8.3 G condition (Ctrl, n = 6; TEA, n = 5; TEA+MDL, n = 5). *P<0.05, ^†^P<0.05.

K_ATP_ channel is a key player in beta cells linking increased glucose metabolism to insulin secretion. It has been well known that blockade of K_ATP_ channels evokes glucose-independent insulin secretion, i.e., no matter at low or high glucose conditions, applying K_ATP_ channel blockers always stimulate insulin secretion. On the contrary, the major functional characterization of K_V_ inhibition is to induce glucose-dependent insulin secretion, i.e., K_V_ inhibition-stimulated insulin secretion only occurs at high glucose conditions. Here, we found at 8.3 mmol/l glucose, 10 µmol/l MDL-12,330A significantly increased insulin secretion(Fig. 6A), and 5 µmol/l MDL-12,330A accordingly showed tendency to increase insulin secretion considering 5 µmol/l MDL-12,330A only inhibit 14% of K_V_ currents (Fig. 6A and 4B). Of note, at 2.8 mmol/l glucose, both concentration of MDL-12,330A had no effect on insulin secretion. Furthermore, our data showed that TEA, a potent K_V_ blocker, stimulated insulin secretion only at high glucose (8.3 mmol/l), but not at low glucose (2.8 mmol/l), and MDL-12,330A had no further effect on insulin secretion in the presence of TEA (Fig. 6B). These results imply that K_V_ channels rather than K_ATP_ channels are involved in the MDL-12,330A effects on insulin secretion.

### MDL-12,330A Increases [Ca^2+^]_i_ Level

**Figure 7:**
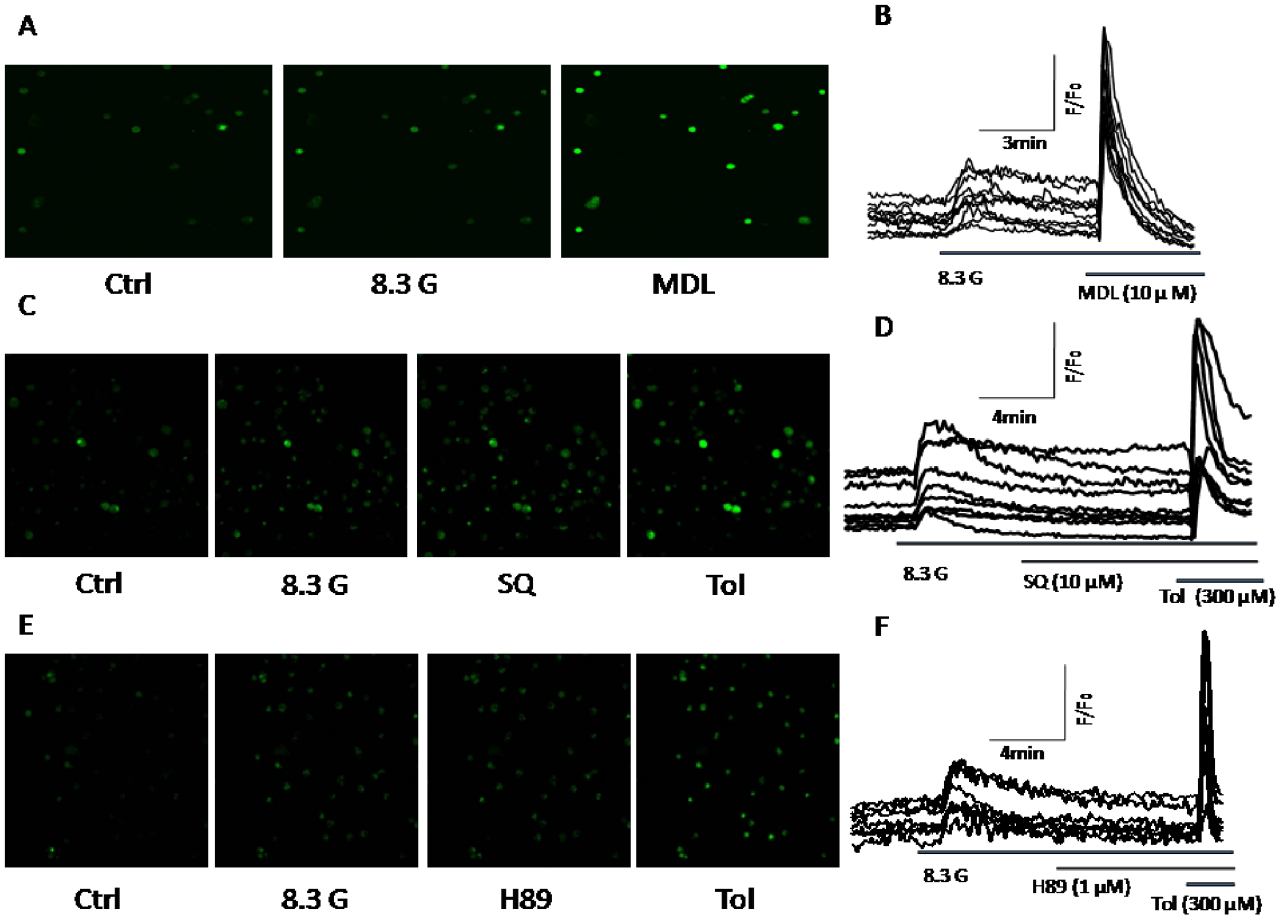
MDL-12,330A increases [Ca^2+^]_i_ level. [Ca^2+^]_i_ was measured from beta cells loaded with Fluo 4-AM using a laser scanning confocal microscope at room temperature. The ratio of fluorescence change F/F_0_ was plotted to represent the change in [Ca^2+^]_i_ levels. A, B. cells were treated with 10 µM MDL-12,330A (MDL, n = 10) in the presence of 8.3 mM glucose (8.3G). C, D. Cells were treated with 10 µM SQ 22536 (SQ, n = 9) in the presence of 8.3 G. E,F. cells were treated with 1 µM H89 (n = 8) in the presence of 8.3 G. As a positive control, Tolbutamide (Tol, 300 µM) was applied after SQ 22536 or H89 treatments.

The elevation of [Ca^2+^]_i_ is essential for triggering beta cell exocytosis. Since it has been demonstrated that blockade of K_V_ channels not only prolongs APD, but also augments [Ca^2+^] [11], [12], [19]_i_, we determined the action of MDL-12,330A on [Ca^2+^]_i_ in pancreatic β-cells using OLYMPUS FV1000 (Inverted microscope IX81) confocal microscope at room temperature. As shown in Figure. 7A and B, increase of glucose from 2.8 mmol/l to 8.3 mmol/l raised [Ca^2+^]_i_, and addition of MDL-12,330A (n = 10) resulted in further elevation of [Ca^2+^]_i_. However, SQ 22536 (n = 9) as well as H89 (n = 8) had no effects on [Ca^2+^]_i_ (Fig. 7D and F). To make sure the cells tested were physiological functional, tolbutamide (300 µmol/l) was applied as a positive control after SQ 22536 or H89 treatments, and the cells showed good response to tolbutamide (Fig. 7D and F ). Combined with the Ca^2+^ channel results showed above, these findings indicate that the effect of MDL-12,330A on [Ca^2+^]_i_ in beta cells is likely mediated by the inhibition of K_V_ channels rather than the modulation of Ca^2+^ channel inherent properties, and the underlying mechanism is not related to AC/cAMP signaling pathway.

## Discussion

MDL-12,330A and SQ 22536 are two structurally different AC inhibitors, which have been widely used in advancing our understanding of the biological functions of AC enzymes [9]. MDL-12,330A inhibits cAMP augmentation induced by GLP-1 in pancreatic beta cells [20], and similar effects for SQ 22536 on cAMP accumulation were also observed in insulin secreting INS-1 cells [21]. Here we show that forskolin-induced cAMP elevation was similarly inhibited by MDL-12,330A and SQ 22536, demonstrating that the two compounds play similar role in the regulation of AC/cAMP signaling. However, in this study, we found MDL-12,330A exerted very different effects from SQ 22536 on beta cell K_V_ channel, AP profile and insulin secretion.

K_V_ channel is a pivotal player in regulating beta cell electrical excitability by repolarizing action potentials. Both pharmacological inhibition or genetic ablation of K_V_ channels have provided evidences that reduction of K_V_ currents prolongs APD, resulting in elevated [Ca^2+^]_i_ level, and potentiated insulin secretion [11], [12], [19]. In the present study, our data demonstrate that MDL-12,330A could potently block K_V_ channels, and we show that MDL-12,330A modulates the excitability of rat beta cells in a manner consistent with the previously reported K_V_ current inhibition [11], [12], [19].

Action potentials were elicited in the current-clamp mode by applying 4 ms, 150 pA current injections in this study. The result revealed that MDL-12,330A significantly prolonged APD, the underlying mechanism could be attributable to the blockade of K_V_ channels since MDL-12,330A has no effect on Ca^2+^ channels. In concert with previous functional studies regarding K_V_ inhibition in pancreatic beta cells, our findings clearly demonstrate that prolongation of APD following K_V_ inhibition by MDL-12,330A led to an increased insulin secretion as a result of increase in [Ca^2+^]_i_ level. The increase of [Ca^2+^]_i_ in response to MDL-12,330A represents the removal of the Ca^2+^ entry limitation from K_V_ activation.

[Ca^2+^]_i_ in beta cells was increased by MDL-12,330A but not by SQ 22536, suggesting that the potentiated insulin secretion by MDL-12,330A does not result from AC inhibition. This conclusion is supported by the fact that the K_V_ current inhibition and APD prolongation by MDL-12,330A was not shared by SQ 22536. Furthermore, the conclusion is also supported by the following observations from this study. First, inhibition of PKA and Epac, the downstream effectors of AC/cAMP signaling, did not affect K_V_ channels. Second, inhibition of PKA and Epac did not influence insulin secretion from rat islets. These findings provide evidences that MDL-12,330A and SQ 22536 indeed have different biological effects on pancreatic beta cells, demonstrating that MDL-12,330A has a non-specific effect on K_V_ channels via an AC-independent mechanism. It is worthy to note that in pituitary cells, MDL-12,330A has been found to inhibit Ca^2+^ channels [22]; and in retinal Müller glia, the nonspecific effect of MDL-12,330A on glycine transport, which is independent of AC inhibition, has also been reported [23]. Considering the significance of ACs in cellular functions, the information about the non-specific actions of MDL-12,330A is necessary for the right interpretation of the experimental results using MDL-12,330A.

In summary, we found that MDL-12,330A, which is widely used as a “selective” AC inhibitor, inhibits K_V_ currents in rat pancreatic beta cells. The K_V_ channel inhibition rather than AC antagonism by MDL-12,330A prolongs APDs, thereby enhancing [Ca^2+^]_i_, and finally leading to potentiation of insulin secretion. The ubiquitous nature of K_V_ channels and the observation that MDL-12,330A appears to block their currents extensively, combined with the fact that numerous AC/cAMP related studies have used this compound, emphasize the importance of these findings. Future studies using MDL-12,330A will need to consider the effects of this agent on K^+^ currents and membrane action potentials.
